# Mortality and Function After Widowhood Among Older Adults With Dementia, Cancer, or Organ Failure

**DOI:** 10.1001/jamanetworkopen.2024.32979

**Published:** 2024-09-12

**Authors:** Rebecca Rodin, Alexander K. Smith, Edie Espejo, Siqi Gan, W. John Boscardin, Lauren J. Hunt, Katherine A. Ornstein, R. Sean Morrison

**Affiliations:** 1 Brookdale Department of Geriatrics and Palliative Medicine, Icahn School of Medicine at Mount Sinai, New York, New York; 2 Division of Geriatrics, University of California, San Francisco; 3 Northern California Institute for Research and Education, San Francisco; 4 Department of Epidemiology and Biostatistics, University of California, San Francisco; 5 Department of Physiological Nursing, University of California, San Francisco; 6 Philip R. Lee Institute for Health Policy Studies, University of California, San Francisco; 7 Global Brain Health Institute, University of California, San Francisco; 8 School of Nursing, Johns Hopkins University, Baltimore, Maryland; 9 James J. Peters Department of Veterans Affairs Medical Center, Bronx, New York

## Abstract

**Importance:**

The widowhood effect, in which mortality increases and function decreases in the period following spousal death, may be heightened in older adults with functional impairment and serious illnesses, such as cancer, dementia, or organ failure, who are highly reliant on others, particularly spouses, for support. Yet there are limited data on widowhood among people with these conditions.

**Objective:**

To determine the association of widowhood with function and mortality among older adults with dementia, cancer, or organ failure.

**Design, Setting, and Participants:**

This longitudinal cohort study used population-based, nationally representative data from the Health and Retirement Study database linked to Medicare claims from 2008 to 2018. Participants were married or partnered community-dwelling adults aged 65 years and older with and without cancer, organ failure, or dementia and functional impairment (function score <9 of 11 points), matched on widowhood event and with follow-up until death or disenrollment. Analyses were conducted from September 2021 to May 2024.

**Exposure:**

Widowhood.

**Main Outcomes and Measures:**

Function score (range 0-11 points; 1 point for independence with each activity of daily living [ADL] or instrumental activity of daily living [IADL]; higher score indicates better function) and 1-year mortality.

**Results:**

Among 13 824 participants (mean [SD] age, 70.1 [5.5] years; 6416 [46.4%] female; mean [SD] baseline function score, 10.2 [1.6] points; 1-year mortality: 0.4%) included, 5732 experienced widowhood. There were 319 matched pairs of people with dementia, 1738 matched pairs without dementia, 95 matched pairs with cancer, 2637 matched pairs without cancer, 85 matched pairs with organ failure, and 2705 matched pairs without organ failure. Compared with participants without these illnesses, widowhood was associated with a decline in function immediately following widowhood for people with cancer (change, −1.17 [95% CI, −2.10 to −0.23] points) or dementia (change, −1.00 [95% CI, −1.52 to −0.48] points) but not organ failure (change, −0.84 [95% CI, −1.69 to 0.00] points). Widowhood was also associated with increased 1-year mortality among people with cancer (hazard ratio [HR], 1.08 [95% CI, 1.04 to 1.13]) or dementia (HR, 1.14 [95% CI, 1.02 to 1.27]) but not organ failure (HR, 1.02 [95% CI, 0.98 to 1.06]).

**Conclusions and Relevance:**

This cohort study found that widowhood was associated with increased functional decline and increased mortality in older adults with functional impairment and dementia or cancer. These findings suggest that persons with these conditions with high caregiver burden may experience a greater widowhood effect.

## Key Points

**Question:**

What is the association of widowhood with function and mortality among older adults with functional impairment and cancer, dementia, or organ failure?

**Findings:**

In this cohort study including 13 824 participants in the Health and Retirement Study, widowhood was associated with functional decline and increased 1-year mortality in functionally impaired older adults with dementia and cancer.

**Meaning:**

These findings suggest that the widowhood experience may impair function and increase mortality in older adults with serious illness, such as dementia and cancer, who rely on others for caregiving support.

## Teaser

This cohort study assesses the association of widowhood among older adults with functional impairment with or without cancer, dementia, or organ failure.

## Introduction

Widowhood is associated with subsequent decline in function and increases in mortality. This widowhood effect may be heightened in people with serious illness whose spouses typically provide considerable caregiving support.^1,2^ Such support may include treatment navigation, nursing, personal hygiene, grocery shopping and meal preparation, transportation, and medical decision-making, in addition to spousal financial, emotional, and social support.^1,3^ Loss of support could affect the functional trajectory and survival of individuals with conditions such as dementia, cancer, and chronic organ failure—the leading causes of US deaths prior to COVID-19.^4^ Widowhood can be conceptualized as an acute social disruptive event that can affect health outcomes, such as mortality and function. However, there are limited data on widowhood in individuals who are seriously ill.

The purpose of this study was to determine whether widowhood is associated with decline in function and increased mortality among older adults with functional impairment and cancer, dementia, or organ failure. These illnesses represent 3 archetypal serious illness trajectories^4,5^: (1) steady progression followed by rapid functional decline before death (cancer); (2) gradual functional decline with episodes of acute decompensation, followed by partial recovery and a sudden, less predictable death (organ failure); and (3) slow, steady functional decline with persistent and prolonged disability and difficult prognostication (dementia). These illness models impose large health care system burdens^6^ and spousal caregiving may be protective against adverse outcomes.^7,8,9,10^

## Methods

### Study Design and Sample

This cohort study was approved by the Icahn School of Medicine at Mount Sinai and University of California, San Francisco, institutional review board with informed consent exemptions because the study team had no contact with the participants, nor were we permitted access to participants’ contact information. This report adhered to the Strengthening the Reporting of Observational Studies in Epidemiology (STROBE) reporting guideline.

We used data from 2008 to 2018 data from The Health and Retirement Study (HRS), a nationally-representative, longitudinal study of US adults ages 50 years and older. The HRS includes participant interviews and links to Medicare claims.^11^ Interviews are conducted every 2 years until death or disenrollment, and replenishment cohorts are added every 6 years. Proxy respondents, typically next of kin, are interviewed if participants are unable to complete an interview and following their death (exit interviews). The HRS has 85% to 90% response rates.

We included 13 862 community-residing (ie, not in a nursing home) participants ages 65 years and older interviewed between 2008 to 2018 who were married or cohabiting with a partner at their first eligible interview. We identified people with diagnoses of dementia, cancer, or organ failure (respiratory or heart) and functional impairment. We defined functional impairment as requiring assistance with at least 2 of any instrumental activities of daily living (IADLs) or activities of daily living (ADLs).^12^ This function criterion was set to identify people with serious illness who are likely to require caregiving support but who are still early enough in their illness course for widowhood to impact their illness trajectory. While differences exist between ADLs and IADLs, we incorporated deficits in either of these domains to account for the significant heterogeneity of functional decline among individuals who are seriously ill (eg, some lose independence with an ADL before an IADL, and vice-versa).^13,14^

We used a validated algorithm to define dementia status,^15^ which estimates the probability of a participant having dementia at each HRS interview using that interview’s cognitive and functional data. We used a probability cutoff of more than 0.5 (90% specificity, 78% sensitivity for identifying dementia).^15^ Within our cohort, 1422 participants developed dementia, of whom 319 required assistance with at least 2 ADLs or IADLs; 1738 people never developed dementia.

Cancer status was defined using HRS survey responses, which have a 72.9% sensitivity and 96.3% specificity.^16^ Within our cohort, 487 people developed cancer, of whom 85 had at least 2 ADL or IADL limitations; 2673 people never developed cancer (eAppendix 1 in Supplement 1). Organ failure (congestive heart failure or chronic nonasthmatic respiratory disease) was also defined using survey responses, which has a 58% sensitivity and 93% specificity for heart failure and is used as the HRS-equivalent criterion standard questionnaire for chronic bronchitis or emphesyma.^17,18,19,20^ We limited the organ failure sample to cardiac or nonasthmatic lung disease because they are among the most common and lethal forms of organ failure in the US^21^ and are readily identifiable and validated in the HRS questionnaire. Within our cohort, 455 people developed organ failure, of whom 85 had at least 2 ADL or IADL limitations; 2705 people never developed organ failure.

In post hoc analyses, we combined people from the cancer, dementia, and organ failure cohorts into an any serious illness cohort, which we compared with people without any of these illnesses. There were 1907 people who developed any serious illness, of whom 375 had at least 2 ADL or IADL limitations, compared with a no serious illness cohort of 2785 participants.

### Cohort Matching

Within each illness cohort, we identified individuals who experienced widowhood among unique participants using the first occurring widowhood event. We matched participants with an event to those without at each wave by calculating propensity scores using covariates of age, gender, illness status (cancer, dementia, or organ failure), number of comorbidities, and education level and then using propensity scores with exact matching on age, gender, HRS wave, and illness status. For the event group, time zero was the widowhood date. For participants without an event, we calculated a simulated reference event time based on the interval start date and matched-case event time (eFigure 1 in Supplement 1). Participants who experienced an event in a later wave could be used as controls in earlier waves, but their postevent function scores were removed (for function analyses only). Participants with comorbid illness could appear in multiple illness cohorts. Nearly all individuals who experienced widowhood (99.8%) were matched. Matching participants at a real or simulated time zero improves causal inference with observational data by emulating a randomized trial start date and may provide less biased estimates than other time-varying covariate survival analyses that do not use matching.^22,23^

### Measures

#### Widowhood

As in other studies,^24,25^ we identified widowhood using the month and year of the spouse’s death from the National Death Index (NDI), ascertained from HRS exit interviews or respondents’ survey responses. The NDI collects spousal death data only if the participant and their spouse are married or partnered and both are enrolled in HRS. Only 0.9% of participants remarried or repartnered following a widowhood event, and this occurred a mean (SD) of 4.5 (2.4) years following widowhood (ie, near the end or after the 5-year postwidowhood follow-up period). We included remarried and repartnered participants because they had sufficient follow-up before any remarriages or repartnerships occurred.

#### Function

We used HRS core and exit surveys to determine functional limitation using the items related to participants’ ability to perform ADLs and IADLs. As in prior studies,^12^ we defined functional impairment as requiring assistance with at least 2 of any of the 6 ADLs (walking, dressing, bathing, eating, getting into and out of bed, and toileting) or 5 IADLS (preparing a hot meal, shopping for groceries, making telephone calls, using medicines, and managing money). These responses are summed to create a total score of 0 to 11. To better illustrate functional decline, we reverse coded scores so that higher scores represent better function (eAppendix 2 in Supplement 1).

#### Mortality

We measured 1-year mortality using date of death, which was determined by reconciling HRS data, a linkage to the NDI, and Medicare claims. This approach captures nearly all deaths.^26^

#### Other Measures

Patient sociodemographic and clinical characteristics were identified to describe the sample and for matching and adjusting. These included age, gender, education level, smoking status, number of comorbidities, and self-reported race and ethnicity. For race and ethnicity, respondents classified themselves as African American or Black, American Indian, Alaska Native, Native Hawaiian or Pacific Islander, White, or other; and whether they identify as Hispanic (eg, Cuban American, Mexican American, Puerto Rican, or something else). We categorized participants as Hispanic, non-Hispanic Black, or non-Hispanic White. Due to small sample sizes, other racial and ethnic categories are not reported. Race and ethnicity data were collected to describe the sample and for matching and adjusting.

### Statistical Analysis

For function, we used linear regression to estimate pre-event and postevent annual slopes up to 2 waves (ie, 5 years) before and after actual or simulated event times. For those with an actual event, we included an indicator term for immediate change in function at the event time. For mortality, we used a time-to-event analysis using Cox regression to estimate 1-year mortality and hazard ratios (HRs), with censoring at 1 year if death did not occur. We used Kaplan-Meier survival curves to depict results visually.

We conducted sensitivity analyses to strengthen our findings. For function, analyses were (1) additional adjustment for potential confounding variables (age, gender, disease status [eg, cancer status for cancer cohort], number of comorbidities, and wealth quartile), (2) weighting for death and dropout using inverse probability and/or survey weighting, (3) modeling with a time-varying covariate approach, and (4) subgroup analysis among participants with functional disability. For mortality, analyses included additional adjustment for potential confounding variables (same as for function analyses) and weighting for death and dropout using inverse probability and/or survey weighting. Further details are available in eAppendix 3 in Supplement 1.

We present unweighted results for our primary findings because our sample included small subgroups for which weighted estimates may not be stable. However, weighted estimates are presented in our sensitivity analyses.^27^ Analyses were conducted using SAS statistical software version 9.4 (SAS Institute) and Stata statistical software version 18 (StataCorp). *P* values were 2-sided, and statistical significance was set at *P* < .05. Analyses were conducted from September 2021 to May 2024.

## Results

Of 13 824 participants (mean [SD] age, 70.1 [5.5] years; 6416 [46.4%] female) included, 5732 experienced widowhood. Our sample included 329 Hispanic participants (2.1%), 1562 non-Hispanic Black participants (9.9%), and 10 640 non-Hispanic White participants (67.3%). After matching, there were 319 matched pairs of people with dementia, 1738 matched pairs without dementia, 95 matched pairs with cancer, 2637 matched pairs without cancer, 85 matched pairs with organ failure, and 2705 matched pairs without organ failure (Table 1). Widowhood and no widowhood cohort characteristics for each illness were similar after matching, except that there was a smaller proportion of Black people among adults with organ failure and widowhood compared with no widowhood (22.1% vs 11.6%) (Table 1).

**Table 1.** Baseline Characteristics of Participants After Matching^a^

Variable	Participants by serious illness and widowhood status, No. (%)											
	Dementia (n = 4114)				Cancer (n = 5516)				Organ failure (n = 5600)			
	No		Yes		No		Yes		No		Yes	
	No widow (n = 1738)	Widow (n = 1)738	No widow (n = 319)	Widow (n = 319)	No widow (n = 2673)	Widow (n = 2673)	No widow (n = 85)	Widow (n = 85)	No widow (n = 2705)	Widow (n = 2705)	No widow (n = 95)	Widow (n = 95)
Age, mean (SD), y	74.4 (6)	75.8 (6)	80.1 (7)	82.8 (7)	75.9 (7)	77.4 (7)	78.9 (7)	81.6 (7)	76.2 (7)	77.7 (7)	77.5 (7)	79.3 (7)
Gender												
Male	558 (32.1)	558 (32.1)	103 (32.3)	103 (32.3)	841 (31.5)	841 (31.5)	32 (37.6)	32 (37.6)	850 (31.4)	850 (31.4)	34 (35.8)	34 (35.8)
Female	1180 (67.9)	1180 (67.9)	216 (67.7)	216 (67.7)	1832 (68.5)	1832 (68.5)	53 (62.4)	53 (62.4)	1855 (68.6)	1855 (68.6)	61 (64.2)	61 (64.2)
Race and ethnicity^b^												
Hispanic	22 (1.3)	17 (1.0)	11 (3.4)	5 (1.6)	32 (1.2)	31 (1.2)	3 (3.5)	2 (2.4)	33 (1.2)	33 (1.2)	0 (0)	2 (2.1)
Non-Hispanic Black	156 (9.0)	158 (9.1)	51 (16)	44 (13.8)	232 (8.7)	274 (10.3)	14 (16.5)	11 (12.9)	239 (8.8)	278 (10.3)	21 (22.1)	11 (11.6)
Non-Hispanic White	1422 (81.8)	1425 (82.0)	215 (67.4)	235 (73.7)	1832 (80.1)	1832 (80.2)	64 (75.3)	67 (78.8)	1855 (80.4)	1855 (80.3)	65 (68.4)	61 (64.2)
High school or GED education	745 (42.9)	740 (42.6)	182 (57.1)	182 (57.1)	1212 (45.3)	1210 (45.3)	39 (45.9)	38 (44.7)	1206 (44.6)	1201 (44.4)	54 (56.8)	55 (57.9)
Smoking	120 (6.9)	168 (9.7)	15 (5.0)	15 (4.7)	185 (6.9)	228 (8.5)	4 (4.7)	6 (7.1)	163 (6.0)	187 (6.9)	12 (12.6)	16 (16.8)
Comorbidities, mean (SD), No.^c^	0.87 (0.91)	0.88 (0.93)	1.38 (1.01)	1.43 (1.09)	0.75 (0.85)	0.75 (0.86)	2.09 (0.86)	2.24 (0.95)	0.76 (0.84)	0.77 (0.85)	2.03 (0.84)	2.19 (1.10)
Dependent for ADL/IADLs, mean (SD)	0.26 (0.92)	0.27 (0.91)	4.18 (3.01)	4.33 (3.16)	0.58 (1.69)	0.60 (1.72)	3.46 (2.58)	3.60 (3.03)	0.49 (1.56)	0.57 (1.71)	3.82 (2.80)	3.61 (2.78)

Abbreviations: ADL, activities of daily living; GED, general equivalency diploma; IADL, instrumental activities of daily living.

^a^ Participants with a widowhood event were matched to those without the event by first calculating propensity scores with the covariates age, gender, illness status (eg, dementia status for the dementia cohort), number of comorbidities, and education level and then using propensity score matching, with exact matching required for gender, study wave, and illness status. For the widowhood event group, the date of the event was set as time 0. For those who did not have a widowhood event, we calculated a simulated event time based on the interval start date and the matched case event time.

^b^ Due to small sample sizes, other racial and ethnic categories are not reported.

^c^ Comorbidities included heart disease, lung disease, stroke, cancer, arthritis, and diabetes.

### Function and Mortality by Illness Category

**Figure 1.**
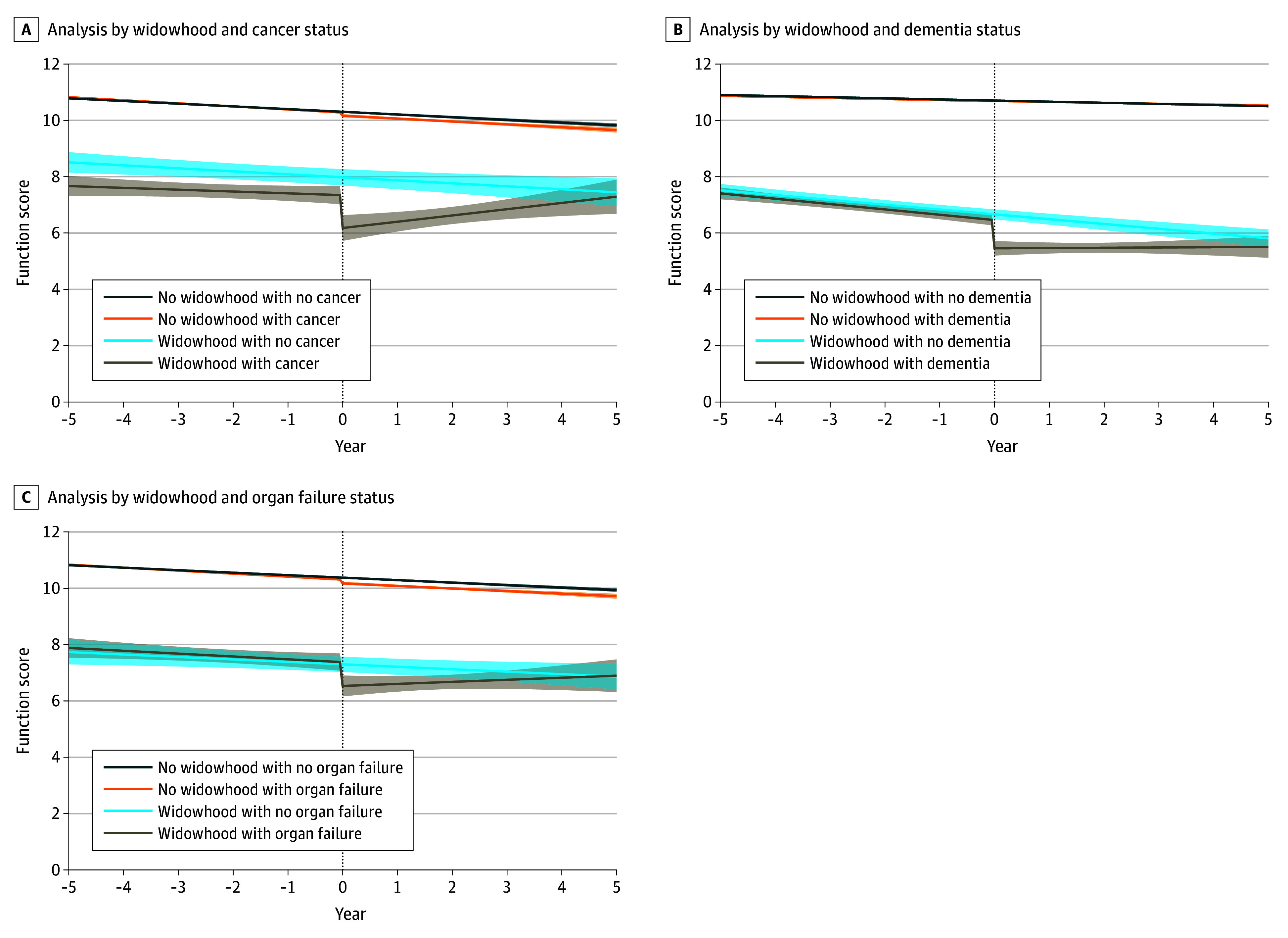
Matched Spline Regression of Function Following Real or Simulated Widowhood Event for People With and Without Cancer, Dementia, or Organ Failure Among people with cancer (A), there were no significant differences in the slopes for those with and without widowhood events (pre-event: *P* = .71; postevent: *P* = .07). Among people with dementia (B), there were no significant differences in the slopes for those with and without widowhood events (pre-event: *P* = .78; postevent: *P* = .11). Among people with organ failure (C), there were no significant differences in the slopes for those with and without widowhood events (pre-event: *P* = .90; postevent: *P* = .35). Dashed line represents time of actual or simulated widowhood event; shading, 95% CI.

**Figure 2.**
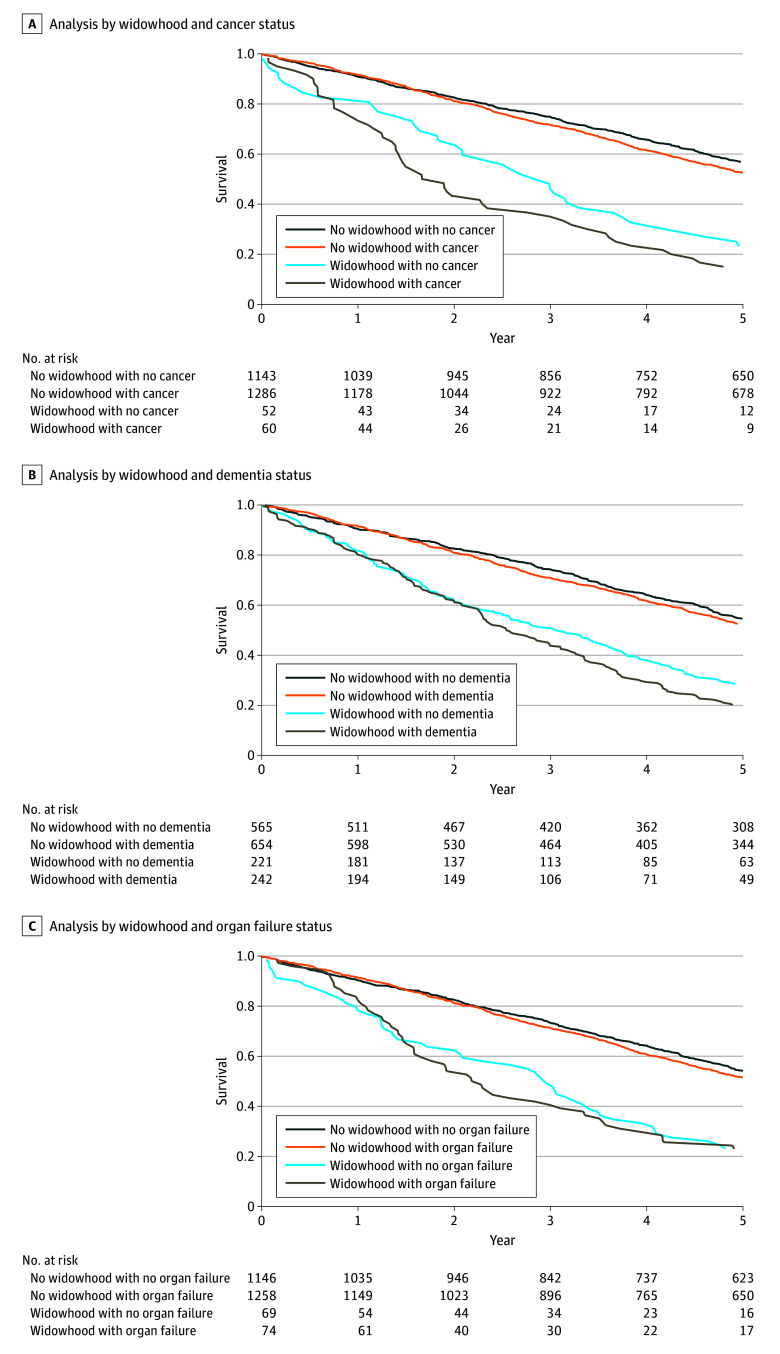
Kaplan-Meier Survival Curves Following Real or Simulated Widowhood Event for People With and Without Cancer, Dementia, or Organ Failure

Older adults with functional impairment and cancer or dementia experienced a decrease in function score of approximately 1 point following widowhood; for those with organ failure, this decrease was not statistically significant. Widowhood was associated with 47% and 14% increases in 1-year mortality for functionally impaired older adults with cancer and dementia, respectively. No significant increase in mortality was observed in participants organ failure (Table 2, Figure 1, and Figure 2).

**Table 2.** Association Between Widowhood and Function and Predicted Mortality for People With and Without Dementia, Cancer, and Organ Failure

Serious illness and widowhood status	Function^a^					Mortality^b^			
	Pre-event		Change at time 0 (95% CI)	Postevent		Estimated 1-y mortality, %	HR (95% CI)		
	Function, mean (SD)	Slope (95% CI)		Slope (95% CI)	Function, mean (SD)		Among all groups	Among participants with illness	
**Dementia**									
No dementia with no widowhood	10.7 (0.8)	−0.04 (−0.05 to −0.03)	NA	−0.04 (−0.05 to −0.36)	10.8 (0.9)	2.2	1 [Reference]		NA
No dementia with widowhood	10.8 (0.7)	−0.04 (−0.05 to −0.02)	0.01 (−0.07 to 0.08)	−0.03 (−0.06 to −0.00)	10.7 (0.9)	2.4	1.09 (1.03 to 1.16)		NA
Dementia with no widowhood	6.6 (2.6)	−0.17 (−0.25 to −0.09)	NA	−0.17 (−0.25 to −0.09)	6.3 (3.6)	4.7	2.22 (2.02 to 2.43)		1 [Reference]
Dementia with widowhood	7.1 (2.6)	−0.19 (−0.30 to 0.08)	−1.00 (−1.52 to −0.48)	−0.19 (−0.30 to −0.09)	5.7 (3.1)	5.4	2.53 (2.30 to 2.77)		1.14 (1.02 to 1.27)
**Cancer**									
No cancer with no widowhood	10.4 (1.5)	−0.10 (−0.11 to −0.08)	NA	−0.10 (−0.11 to −0.08)	10.1 (2.2)	2.2	1 [Reference]		NA
No cancer with widowhood	10.7 (1.2)	−0.11 (−0.13 to −0.10)	−0.11 (−0.21 to −0.02)	−0.10 (−0.15 to −0.06)	10.1 (1.9)	2.4	1.08 (1.04 to 1.13)		NA
Cancer with no widowhood	7.3 (2.4)	−0.11 (−0.23 to −0.02)	NA	−0.11 (−0.23 to 0.02)	7.8 (3.5)	4.8	2.16 (1.82 to 2.56)		1 [Reference]
Cancer with widowhood	7.2 (2.8)	−0.10 (−0.25 to −0.12)	−1.17 (−2.10 to −0.23)	0.22 (−0.12 to 0.56)	6.5 (3.0)	7.0	3.19 (2.72 to 3.73)		1.47 (1.18 to 1.85)
**Organ failure**									
No organ failure with no widowhood	10.5 (1.4)	−0.09 (−0.10 to −0.07)	NA	−0.09 (−0.10 to −0.07)	10.2 (2.0)	2.2	1 [Reference]		NA
No organ failure with widowhood	10.7 (1.1)	−0.11 (−0.12 to −0.09)	−0.13 (−0.23 to −0.03)	−0.09 (−0.13 to −0.05)	10.1 (2.0)	2.2	1.02 (0.98 to 1.06)		NA
Organ failure with no widowhood	6.8 (2.7)	−0.09 (−0.23 to 0.05)	NA	−0.09 (−0.23 to 0.05)	6.6 (3.7)	5.9	2.79 (2.41 to 3.23)		1 [Reference]
Organ failure with widowhood	7.4 (2.6)	−0.10 (−0.28 to 0.08)	−0.84 (−1.69 to 0.00)	−0.07 (−0.24 to 0.38)	6.4 (2.8)	6.6	3.13 (2.72 to 3.61)		1.12 (0.92 to 1.37)

^a^ Function is defined on a reverse-coded on a scale of 0 to 11 that is the sum of requiring assistance in 6 activities of daily living and 5 instrumental activities of daily living. Slope represents the change in function score per year. Function was modeled using linear spline model to estimate pre-event and postevent annual slopes up to 2 waves or 5 years before and after the actual or simulated event times with knots placed at time of event.

^b^ Mortality was modeled using Cox regression with censoring at 1 year if death did not occur.

#### Cancer

Older adults with cancer and functional impairment had larger decreases in function scores at widowhood event (mean [SD] function score: pre-event, 7.2 [2.8] points; postevent, 6.5 [3.0] points; change at event: −1.17 [95% CI, −2.10 to −0.23] points) compared with those without cancer (mean function score: pre-event, 11.0 [95% CI, 11.0 to 11.0] points; postevent, 11.0 [95% CI, 11.0 to 11.0] points; change at event: −0.11 [95% CI, −0.21 to −0.02] points; *P* = .03), as well as a 47% increased 1-year mortality associated with widowhood (mortality rate: 7.0% widowhood vs 4.8% no widowhood; HR, 1.47 [95% CI, 1.18 to 1.85]). Among participants without cancer, widowhood was associated with an 8% increased risk of mortality (mortality rate: 2.4% widowhood vs 2.2% no widowhood; HR, 1.08 [95% CI, 1.04 to 1.13]). (Table 2, Figure 1A, and Figure 2A; eTable 1 and eTable 4 in Supplement 1).

#### Dementia

Older adults with functional impairment and dementia experienced larger decreases in function score at widowhood event (mean [SD] function score: pre-event, 7.1 [2.6] points; postevent, 5.7 [3.1] points; change at event: −1.00 [95% CI, −1.52 to −0.48] points) than those without dementia (mean [SD] function score: pre-event, 10.7 [0.8] points, postevent, 10.8 [0.9] points; change at event: 0.01 [95% CI, −0.07 to 0.08]; *P* < .001). (Table 2 and Figure 1B; eTable 2 in Supplement 1). Widowhood was associated with a 14% increase in 1-year mortality among older adults with functional impairment and dementia (mortality rate: 5.4% widowhood vs 4.7% no widowhood; HR, 1.14 [95% CI, 1.02 to 1.27]), compared with a 9% increase in those without dementia (mortality rate: 2.4% widowhood vs 2.2% no widowhood; HR, 1.09 [95% CI, 1.03 to 1.16]. (Table 2 and Figure 2B; eTable 4 in Supplement 1).

#### Organ Failure

For older adults with functional impairment and organ failure, there was no significant difference in change in function score at widowhood event (mean [SD] function score: pre-event, 7.4 [2.6] points; postevent, 6.4 [2.8] points; change at event: −0.84 [95% CI, −1.69 to 0.00] points) compared with those without organ failure (mean [SD] function score: pre-event, 10.5 [1.4] points; postevent, 10.2 [2.0] points; change at event: −0.13 [95% CI, −0.23 to −0.03]; *P* = .10) (Table 2 and Figure 1C; eTable 3 in Supplement 1). Similarly, widowhood was not significantly associated with increased 1-year mortality among older adults with functional impairment and organ failure (mortality rate: 6.6% widowhood vs 5.9% no widowhood; HR, 1.12 [95% CI, 0.92 to 1.37]) or in those without organ failure (mortality rate: 2.2% widowhood and 2.2% no widowhood; HR, 1.02 [95% CI, 0.98 to 1.06]) (Table 2 and Figure 2C; eTable 4 in Supplement 1).

A post hoc analysis of older adults with functional impairment and any serious illness (cancer, dementia, or organ failure) yielded similar results, with a decrease in function at widowhood event (−0.85 [95% CI, −1.33 to −0.37] points) and 14% increased 1-year mortality (mortality rate: 5.1% widowhood vs 4.5% no widowhood; HR, 1.14; [95% CI, 1.03 to 1.24) (eTable 5, eFigure 2, and eFigure 3 in Supplement 1).

### Sensitivity Analyses

Results for both function and mortality were similar for all sensitivity analyses (eTables 1-4 in Supplement 1). For the function outcome in participants with organ failure, the adjusted and time-varying covariate models were more robust, with confidence intervals below zero (adjusted difference, −0.89 [95% CI, −1.74 to −0.05] points; time-varying covariate, −0.85 [95% CI, −1.07 to −0.64] points). As in our main analysis, no decrease in function was observed following widowhood among older adults with functional impairment but without cancer (difference, 0.31 [95% CI, −0.22 to 0.85] points), without dementia (difference, 1.59 [95% CI, 0.93 to 2.25] points), or without organ failure (0.45 [95% CI, −0.13 to 1.03] points). For mortality, the adjusted model rendered hazards no longer significant for cancer (HR, 1.10 [95% CI, 0.87 to 1.38]) or dementia (HR, 1.10 [95% CI, 0.99 to 1.23]), and the hazard remained not statistically significant in those with organ failure (HR, 0.87 [95% CI, 0.71 to 1.06]).

## Discussion

To our knowledge, this cohort study is the first to examine changes in function associated with spousal loss among older adults with functional impairment and cancer, dementia, or organ failure—individuals who are already at high risk for functional decline. Using longitudinal, nationally representative data, we found that widowhood was associated with a larger decrease in function among those with cancer and dementia compared with those without these conditions and up to a 47% increase in 1-year mortality compared with those who did not experience widowhood. The observed decreases of approximately 1 point of 11 possible points in function score immediately following widowhood are akin to losing the ability to walk independently or use medications without assistance. For participants with organ failure, the change in function score and hazard of mortality were not statistically significant. This study’s strengths include its rigorous methods (eg, matching) and multiple sensitivity analyses to address confounding due to clinical and sociodemographic variables, death or dropout, survey weighting, and modeling approach.

The study findings suggest that older adults with functional impairment and cancer or dementia are at risk of adverse outcomes following widowhood, including functional decline and a marked elevation in the risk of death, in the year after widowhood. The mortality risk may have been highest for participants with cancer in part due to the association of widowhood with functional decline, which is itself strongly correlated with poorer prognosis in cancer.^28^ Prior studies among older adults have shown widowhood to be associated with frailty,^29^ institutionalization,^30^ cognitive decline,^24,31^ and functional impairment.^32,33^ Our study adds new information about the association between widowhood and illness trajectories of people with dementia, cancer, or organ failure.

To our knowledge, this is the first study to find increased mortality associated with widowhood among older adults with dementia, for whom few accurate prognostic tools are available.^34,35^ The finding of increased mortality associated with widowhood among older adults with functional impairment and cancer is consistent with prior work in people with these conditions, showing the highest risk of death is in the first year following spousal death.^36,37,38,39,40,41,42^ The lack of statistically significant difference in mortality observed in participants with organ failure may be due to the small sample size in that widowhood cohort (85 matched pairs) or because we used self-reported diagnoses rather than postmortem cause-of-death data. Individuals in other studies^36,41,42^ who died from cardiovascular events (eg, acute coronary syndrome) following widowhood may have been unaware of their underlying cardiac disease and could have had few to no prior symptoms, unlike our sample who self-reported their diagnosis.

Our study has clinical implications for older adults with functional impairment and serious illnesses, such as dementia, cancer, and organ failure, who have high caregiver needs. People with serious illness often rely on their spouses for prolonged caregiving support,^1^ which may include assistance with daily function, nursing care, management of medications, nutrition, and financial stability, as well as companionship and psychosocial support. Thus, the adverse effects of spousal loss may not only be due to psychological distress and bereavement, but also to shifts in caregiving needs and arrangements, including residential transitions or institutionalization. This information may help patients and families with future care planning and discussions about care goals and treatment choices. This may include increased community or home supports, rehabilitation, or transition to an assisted-living facility if needed. Changes in function may also have important implications for clinical care, such anticancer treatment eligibility, which typically relies on functional status evaluations.^28^ Improving prognostication may be particularly useful in dementia, for which there are few accurate tools to predict changes in function and mortality.^34,35^

### Limitations

This study has limitations. First, we lacked data on illness severity and cancer stage that may influence functional trajectories and mortality. Second, despite matching on multiple clinical and sociodemographic characteristics, there may be bias in our control sample selection that accounts for the observed differences in function and mortality, although adjusting for these characteristics in sensitivity analyses yielded similar results. Similarly, we were unable to account for other sources of caregiving or social support (eg, children, other family members, friends, paid help), which may confound our findings. Third, we cannot exclude the possibility that the association of widowhood with declining function may be due to functional disability itself, rather than the independent effect of serious illness. However, in our sensitivity analyses, we found no association between widowhood and functional decline among older adults with functional impairment but without serious illness. Furthermore, it is not yet known whether the study findings are generalizable to individuals with little to no functional impairment or with other serious illnesses not examined in this study. Future research should explore patterns across other illness types and whether morbidity and mortality following widowhood can be ameliorated by other sources of caregiving support, including community-based long-term or palliative care.

## Conclusions

This cohort study found that widowhood, an acute, socially disruptive event, was associated with significant functional decline and increased mortality in impaired older adults with functional impairment and cancer or dementia. These findings may help guide future care planning and needs assessments following spousal bereavement.

## Notes

Supplement 1. **eAppendix 1.** Additional Detail on Cancer Cohort **eFigure 1.** Matching Approach **eAppendix 2.** Additional Detail on Function Measure **eAppendix 3.** Additional Detail on Sensitivity Analyses and Alternative Approaches **eTable 1.** Sensitivity Analyses for Function Outcome in Those With and Without Cancer **eTable 2.** Sensitivity Analyses for Function Outcome in Those With and Without Dementia **eTable 3.** Sensitivity Analyses for Function Outcome in Those With and Without Organ Failure **eTable 4.** Sensitivity Analyses for Mortality Outcome **eTable 5.** Widowhood, Function, and Mortality in older Adults With Any Serious Illness (Cancer, Dementia, or Organ Failure) **eFigure 2.** Matched Spline Regression of Function Following Real or Simulated Widowhood Event for People With and Without Any Serious Illness (Cancer, Dementia, or Organ Failure) **eFigure 3.** Kaplan-Meier Survival Curve Following Real or Simulated Widowhood Event for People With and Without Any Serious Illness (Cancer, Dementia, or Organ Failure)
eReferences.

Supplement 2.
Data Sharing Statement
