# Supplementary material for: Mortality and Function After Widowhood Among Older Adults With Dementia, Cancer, or Organ Failure
Source: JAMA Netw Open. 2024 Sep 12;7(9):e2432979. doi: 10.1001/jamanetworkopen.2024.32979 (PMC11393717; doi:10.1001/jamanetworkopen.2024.32979)
Supplement: Supplement 1. — eAppendix 1. Additional Detail on Cancer Cohort eFigure 1. Matching Approach eAppendix 2. Additional Detail on Function Measure eAppendix 3. Additional Detail on Sensitivity Analyses and Alternative Approaches eTable 1. Sensitivity Analyses for Function Outcome in Those With and Without Cancer eTable 2. Sensitivity Analyses for Function Outcome in Those With and Without Dementia eTable 3. Sensitivity Analyses for Function Outcome in Those With and Without Organ Failure eTable 4. Sensitivity Analyses for Mortality Outcome eTable 5. Widowhood, Function, and Mortality in older Adults With Any Serious Illness (Cancer, Dementia, or Organ Failure) eFigure 2. Matched Spline Regression of Function Following Real or Simulated Widowhood Event for People With and Without Any Serious Illness (Cancer, Dementia, or Organ Failure) eFigure 3. Kaplan-Meier Survival Curve Following Real or Simulated Widowhood Event for People With and Without Any Serious Illness (Cancer, Dementia, or Organ Failure) eReferences. [file jamanetwopen-e2432979-s001.pdf]

Supplemental Online Content

Rodin R, Smith AK, Espejo E, et al. Mortality and function after widowhood among older adults with dementia, cancer, or organ failure. *JAMA Netw Open*. 2024;7(9):e2432979. doi:10.1001/jamanetworkopen.2024.32979

eAppendix 1. Additional Detail on Cancer Cohort
eFigure 1. Matching Approach
eAppendix 2. Additional Detail on Function Measure
eAppendix 3. Additional Detail on Sensitivity Analyses and Alternative Approaches
eTable 1. Sensitivity Analyses for Function Outcome in Those With and Without Cancer
eTable 2. Sensitivity Analyses for Function Outcome in Those With and Without Dementia
eTable 3. Sensitivity Analyses for Function Outcome in Those With and Without Organ Failure
eTable 4. Sensitivity Analyses for Mortality Outcome
eTable 5. Widowhood, Function, and Mortality in older Adults With Any Serious Illness (Cancer, Dementia, or Organ Failure)
eFigure 2. Matched Spline Regression of Function Following Real or Simulated Widowhood Event for People With and Without Any Serious Illness (Cancer, Dementia, or Organ Failure)
eFigure 3. Kaplan-Meier Survival Curve Following Real or Simulated Widowhood Event for People With and Without Any Serious Illness (Cancer, Dementia, or Organ Failure)
eReferences.

This supplemental material has been provided by the authors to give readers additional information about their work.

© 2024 Rodin R et al. *JAMA Network Open.*

eAppendix 1. Additional detail on cancer cohort

Cancer status was defined using survey responses at HRS interviews, with participants responding 'yes' to the question, '[since your last interview,] has a doctor ever told you that you have cancer or malignant tumor, excluding minor skin cancer?' This method has a sensitivity of 72.9% and a specificity of 96.3%, although it is unable to definitively distinguish incidence cancer cases from prior cases of cancer. However, we estimated incident cases by assessing the proportion of people who reported a new cancer diagnosis after their initial HRS interview, compared to participants who reported an existing cancer diagnosis at the time of their first HRS interview. In our matched sample of older adults with cancer (n=170), 135 (79%) did not have cancer at their first HRS interview (i.e. they initially reported not having cancer, but subsequently reported a new cancer diagnosis in a later HRS interview wave), 9 (5%) had an existing cancer diagnosis at the time of HRS enrollment, and 26 (15%) had an existing cancer diagnosis at the time of enrollment but did not have functional impairment and so were excluded from our cancer cohort. This suggests that the majority of people with cancer in our sample are likely to have incident cases of cancer that developed after their HRS enrollment.

© 2024 Rodin R et al. *JAMA Network Open.*

Figure S1. Matching Approach

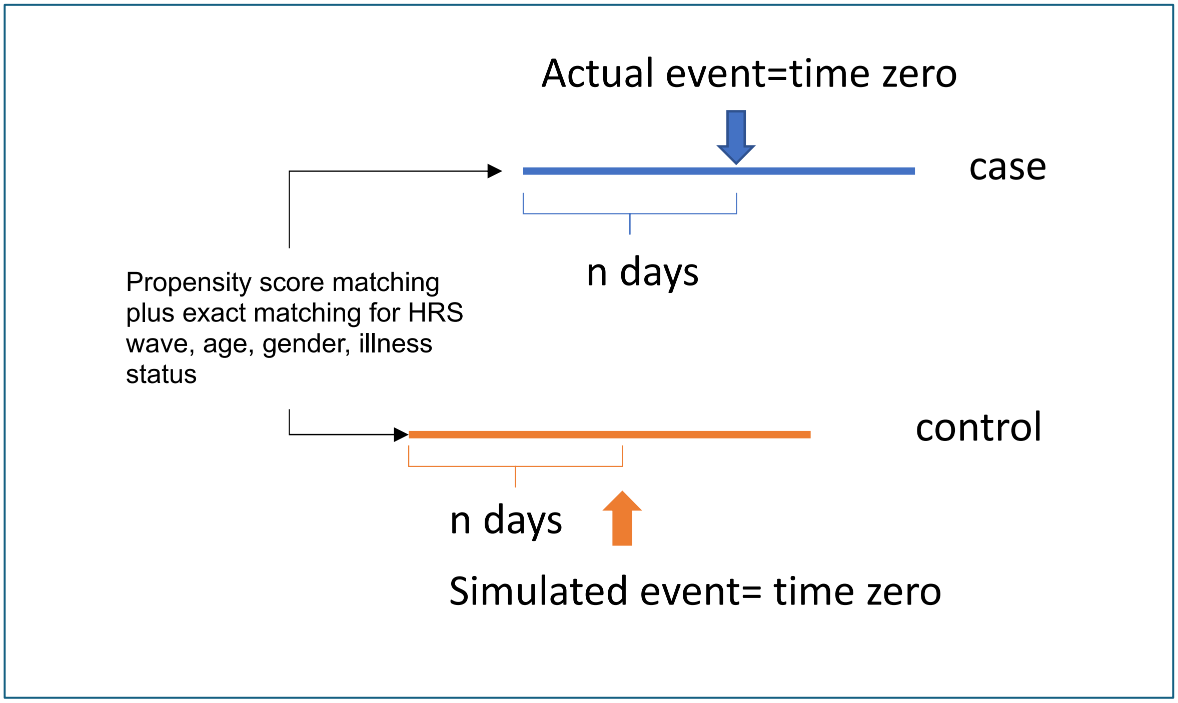

Within each analytic cohort, we identified first cases of widowhood among unique participants. We then matched participants with an event (case) to those without the event (control) at each wave by first calculating propensity scores with the covariates age, gender, number of comorbidities, education level, and illness status (e.g., dementia status for dementia/no dementia cohort). We then used propensity score matching, with exact matching for age, gender, HRS wave, and illness status. For the event group, the date of the event was set as time zero. For those who did not have an event, we calculated a simulated event time based on the interval start date and the matched case event time. This approach is recommended as a method for emulating clinical trials and enhancing causal inference in observational studies.[1,2]

© 2024 Rodin R et al. *JAMA Network Open.*

eAppendix 2. Additional detail on function measure

Function: The HRS core and exit surveys include items on whether participants had difficulty or required assistance with activities of daily living. As in prior studies, we defined As in prior studies,[3] we defined functional impairment as requiring assistance with any 2 of the 6 ADLs (walking, dressing, bathing, eating, getting into and out of bed, and toileting) or 5 IADLS (preparing a hot meal, shopping for groceries, making telephone call, taking medicines, and managing money). These responses are summed to create a total score of 0-11. To better illustrate functional decline, we reverse coded scores so that higher scores represent better function, with 11 indicating needing no assistance and 0 indicating assistance needed on all ADLs and IADLs. For participants who died, we used core interview data when available, otherwise we used exit interview data on function in the last 3 months of life as reported by proxy respondents. In the HRS exit interview, questions regarding ADLs are skipped if the participant stayed in bed for more than 85 days prior to death. For these individuals, we assigned a score of 4 on the 11-item reverse-coded scale. Nursing home residents were also assigned a score of 4 since questions regarding IADLs are skipped for nursing home residents.

© 2024 Rodin R et al. *JAMA Network Open.*

eAppendix 3. Additional detail on sensitivity analyses and alternative approaches

Function:

1) Additional adjustment for potential confounding variables: To account for potential confounding not accounted for by matching, we also ran models including adjustment for variables used in matching (age, gender, disease status [e.g., cancer status for cancer cohort], and number of comorbidities) and wealth quartile.
2) Weighting for death and dropout: We estimated models weighted by the inverse probability of survival (including non-drop out) in order to account for missing outcome data due to death or dropout. We calculated survival probability at each interview by using a multivariable Cox model with the outcome as missing interviews within 2 waves after time 0 due to death or dropout. Subjects who had an exit interview available 2 waves after time 0 were not counted as dead/dropped out.
3) Survey weighting:
    a. We first used Stata svyset command and svy prefix to conduct weighted analysis using HRS complex survey design features, including primary sampling unit (PSU), strata and sampling weights.
    b. We then used a 2-way cluster approach: In our study cohort, a case could also be included as a control before event. In the unweighted analysis presented as main findings, we used clustered robust standard error estimator with clusters for each subject to account for the correlation within subjects. However, robust standard error estimator is not supported in svy prefix. To account for correlation within subjects due to same subject can be both case and control, we created a cluster variable using PSU and strata for each subject and re-ran mix-effect linear regression with sampling weight and clustered robust standard error estimator with cluster created from PSU and strata as well as cluster for subject.
    c. We also performed a separate 2-way cluster approach to account for spousal dyads in our sample: In our cohort study, an individual and his/her spouse may both be included as controls with the potential for one of them to become a case, creating a possibility for their data to be counted three times in our sample. Although this occurred rarely (1 spousal pair in dementia cohort, 5 spousal pairs in each of cancer and organ failure cohorts), we accounted for potential clustering within spousal dyads using clustered robust standard error estimator with clusters for each subject and their spouse. We created a similar cluster variable as above (3b) using PSU and strata for each subject and his/her spouse. We then re-ran mixed effect linear regression with sampling weight and clustered robust standard error estimator with cluster created from PSU and strata as well as cluster for subject and his/her spouse. Weighted results from these three methods (3a, 3b, 3c) were very similar.
4) Weighting for death and drop out and survey weighting: We created a combined weight by multiplying weights constructing in analyses 2 and 3.
5) Time-varying covariate approach: As an alternative to the matched approach used in the primary analyses described previously and in all other sensitivity analyses, we constructed a model that included all participants in our analytic sample with widowhood occurring as a time-varying event. Illness status (e.g., dementia status for dementia/no dementia cohort, cancer status for cancer/no cancer cohort, etc.), was treated as a time-invariant covariate based on status at index interview. The cohort included all individuals included in this study (i.e., not only those who were matched), so has a slightly larger sample size compared to the main analyses. We opted not to use this approach for our primary analyses because of concerns that estimates from this time-varying covariate approach may be biased.[4]
6) Subgroup analysis among all participants with functional disability: To better understand whether the effect of widowhood on function was due to an independent effect of serious illness, rather than functional impairment itself, we conducted a subgroup analysis among all participants with functional disability. In our main analysis, we required that each of the illness cohorts had functional disability (function score ≤9/11) at their index interview, but no such criterion was applied to the cohorts without those illnesses. In this subgroup analysis, we required that all participants had a function score ≤9/11 at their index interview. We then re-matched this subgroup of older adults with functional disability for each of the three serious illnesses and repeated the linear regression described in our main function outcome analysis. We specifically focused on whether functionally impaired older adults without each condition had a drop in

© 2024 Rodin R et al. *JAMA Network Open.*

function score following a widowhood event. The matched subgroups of functionally impaired older adults included 110 without dementia/262 with dementia, 306 without cancer/66 with cancer, and 289 without organ failure/83 with organ failure. These subgroups were limited by small sample size, as there were few widowed participants without each condition who also had functional impairment that were available for matching (84-94% had function scores ≥10/11).

Mortality

1) Additional adjustment for potential confounding variables: same as for function.
2) Survey weighting: We applied survey weighting to account for the Health and Retirement Study's complex survey design and unequal probability of selection. We conducted this analysis based on standard practice recommendations to compare both weighted and unweighted estimates. However, weighted estimates are generally not as impactful for multiple regression analyses and may not be as relevant in analyses of complex subgroups of the original survey.[5]

© 2024 Rodin R et al. *JAMA Network Open.*

Table S1. Sensitivity analyses for function outcome in those with and without cancer

Model	No Cancer (95% CI)					Cancer (95%CI)				
	No Widowhood*		Widowhood			No Widowhood*		Widowhood		
	Pre-slope	Post-slope	Pre-slope	Drop	Post-slope	Pre-slope	Post-slope	Pre-slope	Drop	Post-slope
Unadjusted (Original)	-0.10 (-0.11, -0.08)	-0.10 (-0.11, -0.08)	-0.11 (-0.13, -0.09)	-0.11 (-0.21, -0.02)	-0.10 (-0.15, -0.06)	-0.11 (-0.23, 0.02)	-0.11 (-0.23, 0.02)	-0.06 (-0.25, 0.12)	-1.17 (-2.10, -0.23)	0.22 (-0.12, 0.56)
Adjusted	-0.04 (-0.06, -0.03)	-0.04 (-0.06, -0.03)	-0.06 (-0.08, -0.04)	-0.10 (-0.20, -0.01)	-0.05 (-0.09, 0.00)	-0.08 (-0.21, 0.05)	-0.08 (-0.21 0.05)	-0.05 (-0.24, 0.14)	-1.16 (-2.10, -0.23)	0.26 (-0.08, 0.60)
IPW (un-adjusted)	-0.10 (-0.11, -0.08)	-0.10 (-0.11, -0.08)	-0.11 (-0.13, -0.09)	-0.11 (-0.20, -0.01)	-0.11 (-0.15, -0.06)	-0.11 (-0.23, 0.02)	-0.11 (-0.23, 0.02)	-0.06 (-0.25, 0.13)	-1.14 (-2.06, -0.21)	0.22 (-0.12, 0.56)
Survey weighted svy	-0.10 (-0.11, -0.08)	-0.10 (-0.11, -0.08)	-0.08 (-0.10, -0.07)	-0.16 (-0.27, -0.05)	-0.10 (-0.15, -0.05)	-0.12 (-0.22, -0.02)	-0.12 (-0.22, -0.02)	-0.09 (-0.28, 0.10)	-1.12 (-2.17, -0.07)	0.24 (-0.17, 0.64)
Survey weighted cluster	-0.10 (-0.11, -0.08)	-0.10 (-0.11, -0.08)	-0.08 (-0.10, -0.06)	-0.16 (-0.27, -0.05)	-0.10 (-0.15, -0.05)	-0.12 (-0.23, 0.00)	-0.12 (-0.23, 0.00)	-0.08 (-0.26, 0.09)	-1.13 (-2.13, -0.13)	0.23 (-0.16, 0.62)
Survey weighted, spousal cluster	-0.10 (-0.11, -0.08)	-0.10 (-0.11, -0.08)	-0.08 (-0.10, -0.06)	-0.16 (-0.27, -0.05)	-0.10 (-0.15, -0.05)	-0.12 (-0.23, 0.00)	-0.12 (-0.23, 0.00)	-0.08 (-0.26, 0.09)	-1.13 (-2.13, -0.13)	0.23 (-0.16, 0.62)
IPW + Survey weighted (unadjusted)	-0.09 (-0.11, -0.08)	-0.09 (-0.11, -0.08)	-0.08 (-0.10, -0.06)	-0.16 (-0.26, -0.05)	-0.10 (-0.15, -0.05)	-0.12 (-0.22, -0.02)	-0.12 (-0.22, -0.02)	-0.09 (-0.28, 0.11)	-1.09 (-2.12, -0.07)	0.24 (-0.16, 0.63)
Time-varying covariates	0.00 (0.00, 0.00)	0.00 (0.00, 0.00)	0.00 (0.00, 0.00)	-0.44 (-0.52, -0.37)	-0.01 (-0.01, -0.01)	0.00 (0.00, 0.00)	0.00 (0.00, 0.00)	0.00 (0.00, 0.00)	-0.60 (-0.81, -0.39)	-0.02 (-0.03, -0.01)
Functionally impaired subgroup	-0.21 (-0.29, -0.13)	-0.21 (-0.29, -0.13)	-0.79 (-0.88, -0.70)	0.31 (-0.22, 0.85)	0.00 (-0.25, 0.26)	-0.32 (-0.45, -0.18)	-0.32 (-0.45, -0.18)	-0.23 (-0.52, 0.07)	-0.44 (-1.62, 0.73)	0.03 (-0.44, 0.51)

*'Drop' columns are only presented for event groups, as no change in function is expected in the non-event groups

© 2024 Rodin R et al. *JAMA Network Open.*

Table S2. Sensitivity analyses for function outcome in those with and without dementia

Model	No Dementia (95% CI)					Dementia (95%CI)				
	No Widowhood*		Widowhood			No Widowhood*		Widowhood		
	Pre-slope	Post-slope	Pre-slope	Drop	Post-slope	Pre-slope	Post-slope	Pre-slope	Drop	Post-slope
Unadjusted (Original)	-0.04 (-0.05, -0.03)	-0.04 (-0.05, -0.03)	-0.04 (-0.05, -0.02)	0.01 (-0.07, 0.08)	-0.03 (-0.06, 0.00)	-0.17 (-0.25, -0.09)	-0.17 (-0.25, -0.09)	-0.19 (-0.30, -0.08)	-1.00 (-1.52, -0.48)	0.01 (-0.20, 0.22)
Adjusted	-0.04 (-0.05, -0.03)	-0.02 (-0.04, -0.01)	-0.02 (-0.04, -0.01)	0.01 (-0.06, 0.09)	-0.02 (-0.05, 0.01)	-0.16 (-0.24, -0.08)	-0.16 (-0.24, -0.08)	-0.18 (-0.29, -0.06)	-1.00 (-1.51, -0.48)	0.02 (-0.19, 0.22)
IPW (un-adjusted)	-0.04 (-0.05, -0.03)	-0.04 (-0.05, -0.03)	-0.04 (-0.05, -0.02)	0.01 (-0.06, 0.08)	-0.04 (-0.07, 0.00)	-0.17 (-0.24, -0.09)	-0.17 (-0.24, -0.09)	-0.18 (-0.30, -0.07)	-0.98 (-1.49, -0.47)	0.00 (-0.20, 0.21)
Survey weighted svy	-0.04 (-0.05, -0.03)	-0.04 (-0.05, -0.03)	-0.03 (-0.04, -0.02)	-0.01 (-0.09, 0.07)	-0.03 (-0.06, 0.00)	-0.22 (-0.30, -0.13)	-0.22 (-0.30, -0.13)	-0.13 (-0.26, 0.01)	-1.29 (-1.97, -0.61)	0.02 (-0.23, 0.27)
Survey weighted cluster	-0.04 (-0.05, -0.03)	-0.04 (-0.05, -0.03)	-0.03 (-0.05, -0.02)	-0.01 (-0.08, 0.06)	-0.03 (-0.06, 0.00)	-0.22 (-0.31, -0.12)	-0.22 (-0.31, -0.12)	-0.13 (-0.26, 0.01)	-1.29 (-1.99, -0.60)	0.02 (-0.21, 0.25)
Survey weighted, spousal cluster	-0.04 (-0.05, -0.03)	-0.04 (-0.05, -0.03)	-0.03 (-0.05, -0.02)	-0.01 (-0.08, 0.06)	-0.03 (-0.06, 0.00)	-0.22 (-0.31, -0.12)	-0.22 (-0.31, -0.12)	-0.13 (-0.26, 0.01)	-1.29 (-1.99, -0.60)	0.02 (-0.21, 0.25)
IPW + Survey weighted (unadjusted)	-0.04 (-0.05, -0.03)	-0.04 (-0.05, -0.03)	-0.03 (-0.05, -0.02)	0.00 (-0.08, 0.07)	-0.04 (-0.07, 0.00)	-0.22 (-0.30, -0.13)	-0.22 (-0.30, -0.13)	-0.12 (-0.25, 0.01)	-1.25 (-1.92, -0.58)	0.01 (-0.24, 0.25)
Time-varying covariates	0.00 (0.00, 0.00)	0.00 (0.00, 0.00)	0.00 (0.00, 0.00)	-0.14 (-0.22, -0.05)	0.00 (0.00, 0.00)	0.00 (0.00, 0.00)	0.00 (0.00, 0.00)	0.00 (0.00, 0.00)	-0.96 (-1.08, -0.83)	0.00 (-0.01, 0.00)
Functionally impaired subgroup	-0.14 (-0.26, -0.01)	-0.14 (-0.26, -0.01)	-0.51 (-0.60, -0.43)	1.59 (0.93, 2.25	-0.10 (-0.26, -0.01)	-0.23 (-0.34, -0.12)	-0.23 (-0.45, 0.25)	-0.38 (-0.52, -0.23)	-0.60 (-1.18, -0.01)	0.13 (-0.13, 0.39)
*'Drop' columns are only presented for event groups, as no change in function is expected in the non-event groups										

© 2024 Rodin R et al. *JAMA Network Open.*

Table S3. Sensitivity analyses for function outcome in those with and without organ failure

Model	No Organ Failure (95% CI)					Organ Failure (95%CI)				
	No Widowhood*		Widowhood			No Widowhood*		Widowhood		
	Pre-slope	Post-slope	Pre-slope	Drop	Post-slope	Pre-slope	Post-slope	Pre-slope	Drop	Post-slope
Unadjusted (Original)	-0.09 (-0.10, -0.08)	-0.09 (-0.10, -0.08)	-0.11 (-0.12, -0.09)	-0.13 (-0.23, -0.03)	-0.09 (-0.13, -0.05)	-0.09 (-0.23, 0.05)	-0.09 (-0.23, 0.05)	-0.10 (-0.28, 0.08)	-0.84 (-1.69, 0.00)	0.07 (-0.24, 0.38)
Adjusted	-0.04 (-0.05, -0.03)	-0.04 (-0.05, -0.03)	-0.06 (-0.07, -0.04)	-0.12 (-0.22, -0.02)	-0.04 (-0.08, 0.00)	-0.05 (-0.19, 0.09)	-0.05 (-0.19, 0.09)	-0.06 (-0.24, 0.12)	-0.89 (-1.74, -0.05)	0.11 (-0.20, 0.41)
IPW (un-adjusted)	-0.09 (-0.10, -0.08)	-0.09 (-0.10, -0.08)	-0.11 (-0.12, -0.09)	-0.13 (-0.23, -0.03)	-0.09 (-0.14, -0.05)	-0.09 (-0.20, 0.03)	-0.09 (-0.20, 0.03)	-0.10 (-0.28, 0.08)	-0.81 (-1.66, 0.04)	0.06 (-0.24, 0.37)
Survey weighted svy	-0.08 (-0.09, -0.07)	-0.08 (-0.09, -0.07)	-0.08 (-0.10, -0.06)	-0.18 (-0.29, -0.08)	-0.09 (-0.13, -0.04)	-0.17 (-0.33, -0.01)	-0.17 (-0.33, -0.01)	-0.16 (-0.41, 0.09)	-0.68 (-1.85, 0.48)	0.03 (-0.39, 0.44)
Survey weighted cluster	-0.08 (-0.10, -0.07)	-0.08 (-0.10, -0.07)	-0.08 (-0.10, -0.06)	-0.18 (-0.29, -0.07)	-0.09 (-0.13, -0.04)	-0.17 (-0.37, 0.02)	-0.17 (-0.37, 0.02)	-0.16 (-0.41, 0.08)	-0.69 (-1.90, 0.53)	0.03 (-0.40, 0.45)
Survey weighted, spousal cluster	-0.08 (-0.10, -0.07)	-0.08 (-0.10, -0.07)	-0.08 (-0.10, -0.06)	-0.18 (-0.29, -0.07)	-0.09 (-0.13, -0.04)	-0.17 (-0.37, 0.02)	-0.17 (-0.37, 0.02)	-0.16 (-0.41, 0.08)	-0.69 (-1.90, 0.53)	0.03 (-0.40, 0.45)
IPW + Survey weighted (unadjusted)	-0.08 (-0.09, -0.07)	-0.08 (-0.09, -0.07)	-0.08 (-0.09, -0.06)	-0.18 (-0.28, -0.08)	-0.09 (-0.13, -0.04)	-0.17 (-0.33, -0.01)	-0.17 (-0.33, -0.01)	-0.16 (-0.41, 0.09)	-0.62 (-1.77, 0.53)	0.01 (-0.40, 0.42)
Time-varying covariates	0.00 (0.00, 0.00)	0.00 (0.00, 0.00)	0.00 (0.00, 0.00)	-0.40 (-0.47, -0.32)	-0.01 (-0.01, 0.00)	0.00 (0.00, 0.00)	0.00 (0.00, 0.00)	0.00 (0.00, 0.00)	-0.85 (-1.07, -0.64)	-0.02 (-0.03, -0.01)
Functionally impaired subgroup	-0.25 (-0.33, -0.16)	-0.25 (-0.33, -0.16)	-0.81 (-0.91, -0.72)	0.45 (-0.13, 1.03)	-0.12 (-0.39, 0.15)	-0.19 (-0.34, -0.04)	-0.19 (-0.34, -0.04)	-0.30 (-0.49 (-0.10)	-0.63 (-1.45, 0.18)	0.33 (-0.07, 0.72)

*'Drop' columns are only presented for event groups, as no change in function is expected in the non-event groups

© 2024 Rodin R et al. *JAMA Network Open.*

Table S4. Sensitivity analyses for mortality outcome

	Original			Adjusted			Survey-Weighted		
	1 -Year Mortality, %	Hazard Ratio (95% CI)		1 -Year Mortality, %	Hazard Ratio (95% CI)		1 -Year Mortality, %	Hazard Ratio (95% CI)	
**Dementia**									
No dementia, No widowhood	2.2%	Ref.		2.0%	Ref.		2.2%	Ref.	
No dementia, Widowhood	2.4%	1.09 (1.03, 1.16)		2.2%	1.07 (1.01, 1.14)		2.4%	1.09 (1.03, 1.16)	
Dementia, No widowhood	4.7%	2.22 (2.02, 2.43)	Ref.	3.7%	1.84 (1.67, 2.03)	Ref.	4.7%	2.22 (2.02, 2.43)	Ref.
Dementia, Widowhood	5.4%	2.53 (2.30, 2.77)	1.14 (1.02, 1.27)	4.1%	2.03 (1.84, 2.24)	1.1 (0.99, 1.23)	5.4%	2.53 (2.30, 2.77)	1.14 (1.02, 1.27)
**Cancer**									
No cancer, No widowhood	2.2%	Ref.		2.0%	Ref.		2.2%	Ref.	
No Cancer, Widowhood	2.4%	1.08 (1.04, 1.13)		2.2%	1.06 (1.02, 1.11)		2.4%	1.08 (1.03, 1.13)	
Cancer, No widowhood	4.8%	2.16 (1.82, 2.56)	Ref.	2.8%	1.39 (1.17, 1.65)	Ref.	4.8%	2.16 (1.82, 2.56)	Ref.
Cancer, Widowhood	7.0%	3.19 (2.72, 3.73)	1.47 (1.18, 1.85)	3.1%	1.53 (1.30, 1.80)	1.1 (0.87, 1.38)	7.0%	3.19 (2.72, 3.73)	1.47 (1.18, 1.85)
**Organ Failure**									
No organ failure, No widowhood	2.2%	Ref.		2.0%	Ref.		2.2%	Ref.	
No organ failure, Widowhood	2.2%	1.02 (0.98, 1.06)		2.0%	1.01 (0.97, 1.05)		2.2%	1.02 (0.98, 1.06)	
Organ failure, No widowhood	5.9%	2.79 (2.41, 3.23)	Ref.	3.7%	1.85 (1.60, 2.15)	Ref.	5.9%	2.79 (2.41, 3.23)	Ref.
Organ failure, Widowhood	6.6%	3.13 (2.72, 3.61)	1.12 (0.92, 1.37)	3.2%	1.60 (1.38, 1.86)	0.87 (0.71, 1.06)	6.6%	3.13 (2.72, 3.61)	1.12 (0.92, 1.37)
Abbreviations: CI: confidence interval; Ref: reference									

© 2024 Rodin R et al. *JAMA Network Open.*

Table S5. Widowhood, function, and mortality in older adults with any serious illness (cancer, dementia, or organ failure)

	Function[a,b]			Mortality[c]		
	Pre-event slope (95% CI)	Drop at time 0 (95% CI)	Post-event slope (95% CI)	Predicted 1-year mortality	Hazard Ratio (95% CI)*insert footnote about second HR*	
**Any Illness**						
No illness/No widowhood	-0.03 (-0.04, -0.03)		-0.03 (-0.04, -0.02)	1.6%	Ref.	
No illness/Widowhood	-0.03 (-0.05, -0.02)	0.06 (-0.02, 0.14)	-0.03 (-0.07, -0.01)	1.8%	1.12 (1.04, 1.21)	
Any illness/No widowhood	-0.12 (-0.20, -0.23)		-0.12 (-0.20, -0.04)	4.5%	2.77 (2.51, 3.06)	Ref
Any illness/Widowhood	-0.18 (-0.28, 0.04)	-0.85 (-1.33, -0.14)	0.10 (-0.09, 0.29)	5.1%	3.16 (2.87, 3.49)	1.14 (1.03, 1.26)

CI=Confidence Interval

aFunction was modeled using linear spline model to estimate pre- and post-event annual slopes up to 2-waves or 5 years before/after the actual or simulated event times with knots placed at time of event.

[b]Function is defined on a reverse-coded 0-11 scale that is the sum of requiring assistance in 6 activities of daily living and 5 instrumental activities of daily living. ß represents the change in function score per year.

[c]Mortality was modeled using Cox regression with censoring at 1 year if death did not occur

© 2024 Rodin R et al. *JAMA Network Open.*

Figure S2. Matched spline regression of function following real or simulated widowhood event for people with and without any serious illness (cancer, dementia, or organ failure)

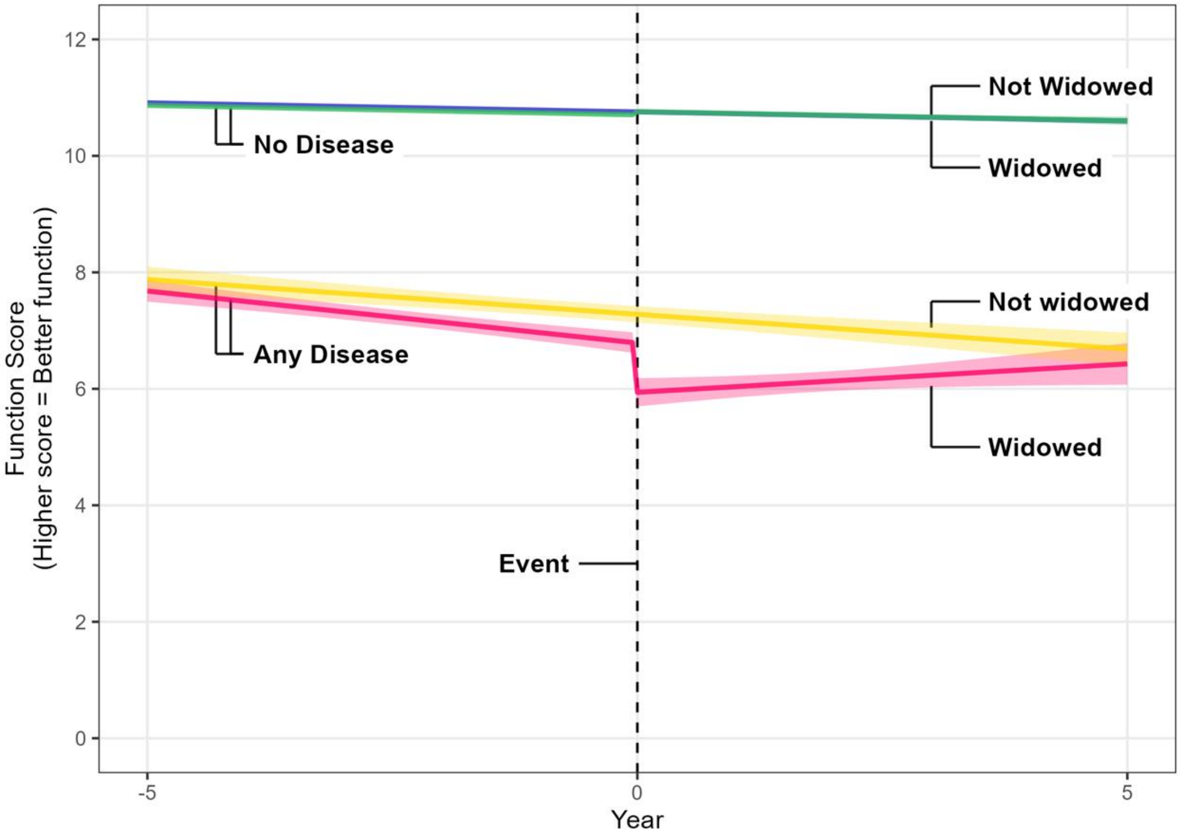

© 2024 Rodin R et al. *JAMA Network Open.*

Figure S3. Kaplan-Meier survival curve following real or simulated widowhood event for people with and without any serious illness (cancer, dementia, or organ failure)

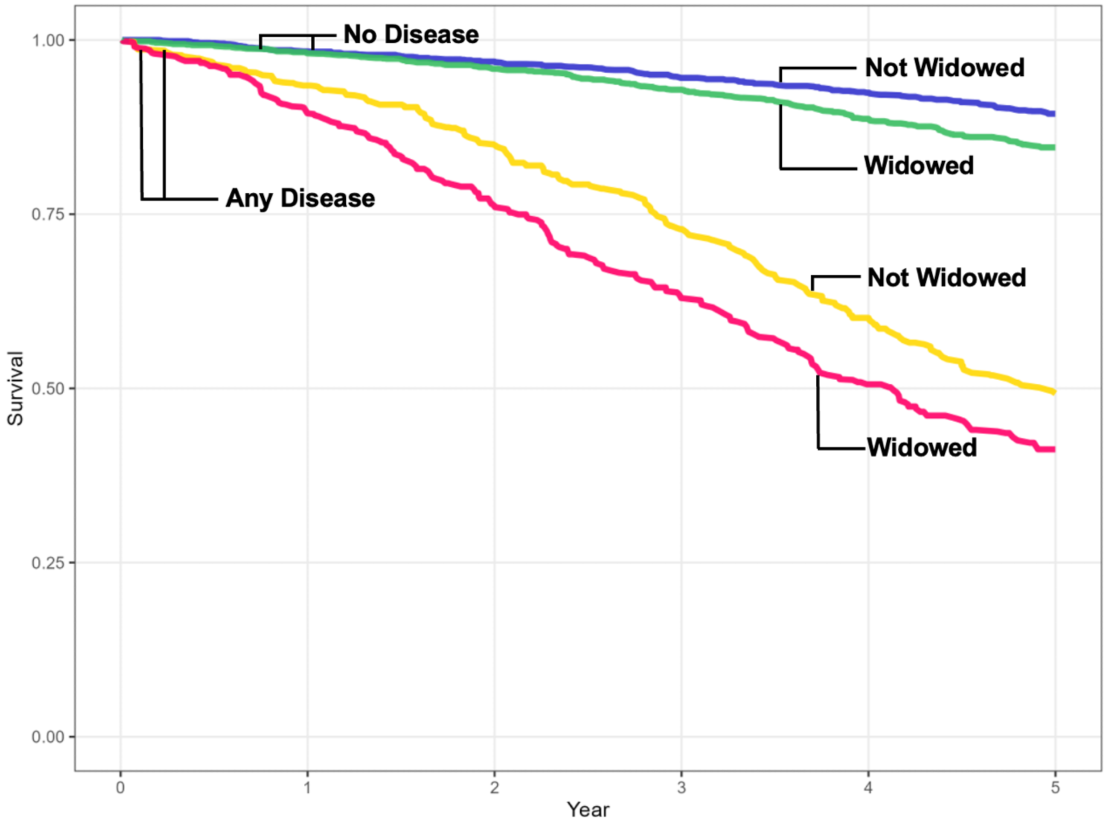

© 2024 Rodin R et al. *JAMA Network Open.*

References

1. Hernán MA, Wang W, Leaf DE. Target Trial Emulation: A Framework for Causal Inference From Observational Data. *JAMA*. 2022;328(24):2446-2447. doi:10.1001/jama.2022.21383
2. Ioannou GN, Bohnert ASB, O'Hare AM, et al. Effectiveness of mRNA COVID-19 Vaccine Boosters Against Infection, Hospitalization, and Death: A Target Trial Emulation in the Omicron (B.1.1.529) Variant Era. *Ann Intern Med*. Dec 2022;175(12):1693-1706. doi:10.7326/m22-1856
3. Iwashyna TJ, Ely EW, Smith DM, Langa KM. Long-term Cognitive Impairment and Functional Disability Among Survivors of Severe Sepsis. *JAMA*. 2010;304(16):1787-1794. doi:10.1001/jama.2010.1553
4. Austin PC, Latouche A, Fine JP. A review of the use of time-varying covariates in the Fine-Gray subdistribution hazard competing risk regression model. *Stat Med*. Jan 30 2020;39(2):103-113. doi:10.1002/sim.8399
5. Si Y, Lee S, Heeringa SG. Population Weighting in Statistical Analysis. *JAMA Internal Medicine*. 2024;184(1):98-99. doi:10.1001/jamainternmed.2023.6300

© 2024 Rodin R et al. *JAMA Network Open.*
