# Supplementary material for: Mortality and Function After Widowhood Among Older Adults With Dementia, Cancer, or Organ Failure
Source: JAMA Netw Open. 2024 Sep 12;7(9):e2432979. doi: 10.1001/jamanetworkopen.2024.32979 (PMC11393717; doi:10.1001/jamanetworkopen.2024.32979)
Supplement: Supplement 2. — Data Sharing Statement [file jamanetwopen-e2432979-s002.pdf]

Data Sharing Statement

Rodin. Mortality and Function After Widowhood Among Older Adults With Dementia, Cancer, or Organ Failure. *JAMA Netw Open*. Published September 12, 2024.
doi:10.1001/jamanetworkopen.2024.32979

Data
Data available: No

Additional Information
Explanation for why data not available: The existing HRS dataset contains data that have already been collected and re-contacting participants is not possible. Access to the data requires the completion of a data use agreement, which prohibits any redistribution of the data to other researchers or attempts to re-identify research participants. Researchers may access the data by requesting it and executing and a data use agreement with CMS.
